# Efficacy and Safety of Oral Fosfomycin-Trometamol in Male Urinary Tract Infections with Multidrug-Resistant Enterobacterales

**DOI:** 10.3390/antibiotics11020198

**Published:** 2022-02-03

**Authors:** Kévin Bouiller, Souheil Zayet, Paul-Emile Lalloz, Anaïs Potron, Vincent Gendrin, Catherine Chirouze, Timothée Klopfenstein

**Affiliations:** 1 Department of Infectious and Tropical Diseases, CHRU, 25000 Besançon, France; kbouiller@chu-besancon.fr (K.B.); paulemile.lalloz@gmail.com (P.-E.L.); catherine.chirouze@univ-fcomte.fr (C.C.); 2 Unité Mixte de Recherche Centre National de la Recherche Scientifique (UMR CNRS) 6249 Chrono-Environnement, University of Bourgogne Franche-Comté, 25000 Besançon, France; 3 Tropical and Infectious Diseases Department, Hospital Nord Franche-Comté, 90400 Trévenans, France; vincent.gendrin@hnfc.fr (V.G.); timothee.klopfenstein@hnfc.fr (T.K.); 4 Bacteriology Laboratory, CHRU, 25000 Besançon, France; apotron@chu-besancon.fr

**Keywords:** fosfomycin, Enterobacterales, male urinary tract infections, prostatitis, efficacy, safety

## Abstract

Background: Antimicrobial drugs to treat male urinary tract infection (UTI) with multidrug-resistant Enterobacterales are limited. We studied oral fosfomycin-trometamol (FT) in this situation. The objective was to assess the clinical cure rate in patients presenting UTIs treated with oral FT. Methods: We conducted a single-center observational retrospective study from January 2017 to August 2018. The primary endpoint was clinical cure; and the secondary endpoints were incidence of recurrences, oral FT safety, and microbiological cure. Results: Sixteen male patients were included, presenting 21 UTI episodes. Fourteen patients (88%) have at least one underlying urologic disorder. We described 4 episodes of acute UTI and 17 episodes of chronic bacterial prostatitis (CBP). Sixteen out of twenty-one Enterobacterales were extended spectrum beta-lactamase (ESBL)-producers and all the patients presented a resistance to fluoroquinolones and trimethoprim/sulfamethoxazole. In acute UTI, the regimen was a daily dose of oral FT for a mean duration of 2.5 weeks (+/−7.0 days). Clinical and microbiological recovery was achieved in all patients, with no recurrence after 5.3 months follow-up on average (+/−10.4 days). In CBP, the regimen was one oral dose of fosfomycin every 24–48 h, for a mean duration of 5.5 weeks/UTI episodes (+/−15.3 days). Clinical and microbiological recovery was found in 16/17 cases. Seven of the twelve patients with CBP had relapsed and 3/12 had had a new episode of infection after an average follow-up of 5.8 months. Only 6/21 of patients presented minor or moderate adverse effects, such as digestive disorders. Conclusions: FT could be an alternative option to carbapenems in the treatment of multidrug-resistant Enterobacterales infections for male UTIs.

## 1. Introduction

The prevalence of male urinary tract infections (UTIs) is estimated to be between 1.5 and 9% in the general population [1,2]. UTIs represent the first site of nosocomial bacterial infections, and the second in the community, in France [3]. The incidence of infections caused by extended-spectrum B-lactamase producing Enterobacterales (ESBL-E) has increased dramatically in French hospitals and *Escherichia coli* strains are the most prevalent species among ESBL-E [3]. This resistance is often accompanied by co-resistance to fluoroquinolones (FQ) and trimethoprim/sulfamethoxazole (SMX-TMP) [4,5,6]. In this situation, the therapeutic choice is limited and often concerns carbapenems, which remain the gold standard for most ESBL-E infections—the consumption of which is increasing worrying worldwide [7]. The ecological impact of this class of antibiotic on the gut microbiota, with the appearance of emerging carbapenemase-producing Enterobacterales (CPE), has dramatic consequences in terms of mortality and cost to society, and must be limited as much as possible [8].

Oral fosfomycin-trometamol (FT) could be an alternative to carbapenems in male ESBL-E UTIs. Regarding the epidemiology of the resistance among ESBL strains, ESBL-E remains susceptible to FT in 90% of cases, and to EBSL *E. coli* in more than 95% of cases [5,9,10,11,12,13,14,15]. Usually in France, FT is indicated as a single oral dose for the first-line treatment of cystitis [1] and is used in combination with intravenous (IV) administration for the treatment of systemic infections. However, it is not currently indicated for the treatment of male UTIs [1]. Only few studies suggested a possible place in this indication [5,12,13,14]. Recent clinical data support efficacy for the treatment of male UTIs with a clinical cure rate of 50 to 77% and a microbiological eradication rate of more than 50% in male UTIs with *E. coli* [2,16,17,18,19]. Pharmacokinetic data show intra-prostatic diffusion at therapeutic rates with a prostate/blood ratio of 60–70% and an intra-prostatic therapeutic concentration 17 h after administration [20]. In addition, oral FT is simple to administer (single dose per day) but needs to be balanced against the risk of drug interactions and adverse effects, which are infrequent [14]. There are fewer safety data for extended treatment of oral FT, but it seems reassuring, even for durations longer than three weeks [18,21]. Its ecological impact is considered to be minor even if it has not really been studied [1,22,23]. Compared to carbapenems, its cost is extremely low [22,23].

In our facility, FT is sometimes used in cases of male UTIs, usually as an alternative to carbapenems in cases of ESBL-E, due to the convenience of administration. We were therefore interested in describing the clinical evolution and safety of male multidrug-resistant Enterobacterales UTI cases treated with oral FT.

## 2. Material and Method

### 2.1. Study Design and Participants

A single-center observational retrospective study was conducted from January 2017 to August 2018. We included all patients with a documented male Enterobacterales UTI susceptible to FT and treated with oral FT—used alone without combination with other active antimicrobials drugs. Exclusion criteria were: children and adolescents (age < 18 years) and patients having received antibiotics (other than oral FT) for which the strain of Enterobacterales was susceptible (excluding molecules with poor prostatic diffusion: amoxicillin-clavulanic acid, cefixime, and nitrofurantoin) [24]. The data were collected from medical records; and, after calling the patient, the attending physician and laboratories if necessary.

The Fosfomycin minimum inhibitory concentrations (MICs) were determined using Etest^®^ (BioMérieux, Marcy-L’Etoile, France) and interpreted according to the EUCAST guidelines. For all strains, Fosfomycin susceptibility was studied using antibiotic disk diffusion testing with a zone diameter breakpoint at 24 mm (EUCAST guidelines). Unfortunately, not all strains were available for MIC determination using Etest^®^.

### 2.2. Definitions

Acute male UTI was defined by:

− The presence of at least one of these symptoms: fever > 38 °C and/or genitourinary symptoms (i.e, suprapubic pain or tenderness, and/or dysuria, and/or urinary frequency, and/or urinary burns, and/or macroscopic haematuria, and/or acute urine retention, and/or pain on the digital rectal examination, and/or confusion).
− And a routine urine culture with leukocyturia > 10/mm³ and bacteriuria > 10³ CFU/mL.
− Chronic bacterial prostatitis (CBP) was defined by the above criteria combined with:
− Clinical signs lasting more than 3 months.
− And/or a history of UTI with the same strain with the same Antimicrobial Susceptibility Testing (AST) in the last three months.

Immunosuppression was defined as: steroids at >20 mg prednisone equivalent per day for ≥14 days before the diagnosis of UTI, chemotherapy within 3 months before the diagnosis of UTI, immuno-modulators, transplantation, and cirrhosis.

Urologic disorders in male patients include benign prostatic hyperplasia, prostatic calcification, urinary tract cancer, vesicoureteral reflux, and bladder control disorders.

Chronic kidney disease (CKD) was defined as an estimated glomerular filtration rate (eGFR) less than 60 mL/min/1.73 m^2^ persisting for three months.

The primary endpoint of the study was clinical cure in patients treated with oral FT, defined by the disappearance under treatment of the clinical signs that led to the diagnosis of male UTI. Thus, failure was defined by the persistence or recurrence of clinical symptoms while being treated or within one week of discontinuing the treatment.

The secondary objectives were to assess:

− Recurrence of UTI, defined as UTI with the same Enterobacterales strain with the same AST, within six months follow-up.
− A new UTI, defined by an UTI to another type of Enterobacterales strain or the same type of Enterobacterales strain but with a different AST (except for fosfomycin resistance).
− Microbiological recovery, defined by a sterile urine culture under treatment and/or within one month after treatment.
− The safety of FT administration. Adverse events (AEs) were classified as serious (requiring hospitalization), severe (requiring discontinuation of oral FT), moderate (requiring symptomatic treatment), and minor (requiring no specific management).

### 2.3. Data Analysis

Concerning the statistical analysis, continuous variables were expressed as mean and standard deviation (SD). Categorical variables were expressed as number (%) and compared by χ^2^ test or Fisher’s exact test. A *p*-value < 0.05 was considered significant. The nonparametric bootstrap method was used to obtain 95% pointwise confidence intervals (95% CI). We used the SPSS v24.0 software (IBM, Armonk, NY, USA).

### 2.4. Ethics Statement Characteristics

Due to the retrospective nature of the study, patients’ consent was not required. We have made sure to keep patients’ data confidential and in compliance with the Declaration of Helsinki.

## 3. Results

### 3.1. Patient Characteristics

Twenty-one episodes of UTI were treated with oral FT in a total of sixteen male patients (Table 1). The mean age was 66.1 years (+/−12.3, (38–83)). The mean Charlson comorbidity index was 4.7 (+/−3.1, (0–10)). Fourteen patients (88%) had at least one underlying urological disorder, four had immunosuppression, and four had CKD.

Fifteen UTI episode strains were *E. coli* (most prevalent species), six were *K. pneumoniae*, sixteen were ESBL, and there were only two strains that were susceptible to penicillins and all of them were resistant to FQ and SMX-TMP. 

### 3.2. Acute Male UTI

Four patients had acute male UTI, of whom three had fever and one had a clinical presentation of acute pyelonephritis with acute kidney failure. Two patients had a C-Reactive Protein (CRP) >150 mg/L (normal range <5 mg/L).

The oral FT regimen was a single dose daily for a mean duration of 2.5 weeks ([1,2,3], +/−7 days). All patients were clinically and microbiologically cured, with no recurrence at six months.

### 3.3. Chronic Bacterial Prostatitis

Twelve patients had a CBP presentation. All of them had urological disorders and four had immunosuppression, while six had a high Charlson comorbidity index >3. The duration of treatment was 5.5 weeks/episode ([3,4,5,6,7,8,9,10,11,12], +/−15.3 days). In 14/17 UTI episodes, the patients had 1 daily dose for the first 3 weeks followed by 1 dose every 48 h.

All patients had clinical and microbiological recovery, except for one patient who had clinical and microbiological failure but the initial MIC of fosfomycin revealed afterwards that the strain was resistant to fosfomycin. Seven out of twelve patients had a recurrence, and three out of twelve patients had a new UTI at six months.

Four patients with recurrent UTI were treated again with oral FT with clinical and microbiological recovery in all cases and no recurrence after an average of 5.5 months ([1,2,3,4,5,6], +/−11.6 days) of follow-up (Table 1).

### 3.4. Fosfomycin-Trometamol Safety

No serious AEs have been attributed to oral FT and no patients have had to stop treatment due to AEs. Diarrhea was the only AE described in 6/21 UTI episodes; 4 patients had minor diarrhea and 2 had moderate diarrhea requiring symptomatic treatment. No other AEs were described in the patients. 

The patients treated with oral FT for more than 3 weeks had no more diarrhea than patients with oral FT less than 3 weeks (respectively 3/14 vs. 3/7, *p* = 0.35). For 4/6 patients who had diarrhea, it had occurred during the daily intake of FT (for the other 2 patients the information could not be specified). The patients with chronic kidney failure did not have more diarrhea than patients with normal kidney function when treated with oral FT (0/6 episodes of UTI in patients with chronic kidney failure vs. 6/15 episodes of UTI in patients with clearance >60 mL/min, *p* = 0.12).

## 4. Discussion

### 4.1. Patient Characteristics

In this study, oral FT was prescribed mostly in cases of ESBL-E CBP with cross-resistance to FQ and SMX-TMP in patients with an underlying urological disorder. The same characteristics are usually described in the literature (Table 2), but with some particularities in our study. Concerning co-morbidities, four patients (25%) were immunocompromised, which is usually not described. Concerning the clinical features, 50% (8/16) of the patients had a fever and seven of eleven patients (64%) had an elevated CRP. However, in France, it is particularly not recommended to prescribe oral FT in the case of a febrile male UTI [25]. Regarding resistances among ESBL strains, 5 out of 21 did not produce ESBL, but all were resistant to FQ and SMX-TMP.

### 4.2. Acute Male UTI 

Oral FT was efficient in all our cases without recurrences after six months’ follow-up. In acute UTIs, shorter treatments were prescribed in medical literature, often 3 doses in total with a clinical cure >70% and a microbiological cure >50% (Table 2) [26,27,28,30,31]. However, most of the cohorts are heterogeneous with a female predominance and there is no information concerning remote monitoring and the presence or absence of recurrence. For example, Senol et al., with a 3-day oral FT regimen (D1, D2, D3), found a similar efficiency compared to carbapenems (meropenem 3 g once a day or daily IV or imipenem cilastatin 2 g per day IV for 14 days) in cases of acute UTI. However, in the FT arm, 14 of 27 patients (52%) were female [29].

Thus, treatment with oral FT appears to be effective in cases of acute male UTI, but data remain heterogeneous. The mean duration of treatment in our study was 2.5 weeks, which may seem long in this situation. However, it should be remembered:

- the lack of data, in particular the absence of a randomized trial against a reference molecule with good prostatic diffusion such as FQ,
- the need for a usually lengthy treatment (≥2 weeks) in male UTIs in the absence of criteria to exclude prostatic damage [1],
- the pharmacokinetics of oral FT in prostate tissue with a concentration rate > MIC (example taken at 4 mg/L) of 80–100% for the first 12 h but <20% at 24 h (modelling concerning the transition zone of the prostate, concentrations even lower for the peripheral zone) [36],
- good safety for the 3 g/24 h dosage.

Currently, a daily dose of 3 g for at least 14 days seems necessary in the case of acute male UTI. In the absence of prostatic damage (in case of a male UTI cystitis like), a spacing of doses and a shorter duration (1 or 3 doses of FT) would probably remain effective. However, as the absence of prostatic damage is not detectable in current practice, there are currently no recommendations in that case [1]. The methodology of our study does not allow us to draw any conclusions. Recently a randomized study in patients with non-febrile male urinary tract infection showed non-inferiority of treatment with ciprofloxacin or SMX-TMP for 7 days versus 14 days [33]. Further randomized studies could answer the question of the value of fosfomycin in this indication.

### 4.3. Chronic Bacterial Prostatitis 

Seventeen episodes of chronic prostatitis were treated with an oral FT regimen of 1 dose/24–48 h for a total mean duration of 5.5 weeks [3,4,5,6,7,8,9,10,11,12]. Clinical and microbiological recovery was achieved in more than 90% of cases.

In the literature, apart from a few cases reported in medical literature, only 2 cohorts of chronic prostatitis with limited numbers of patients (15 and 20) have been treated with FT oral. Karaikos et al. found similar clinical results with 85% recovery in patients presenting with male UTI and treated with an oral FT regimen, which is similar to that of our study (3 g/24 h for 1 week followed by 3 g/48 h for 5 weeks) but only with a 3-month follow-up with no microbiological control [34]; however, there regimen was more economical. Los Arcos et al. showed a recovery at the end of treatment in 93% of patients (14/15) and a recurrence rate of 53% in patients (8/15) after a mean follow-up of 20 months. Microbiological colonization in urine cultures at 6 months was described in 47% of patients (7/15); all these results are close to those found in our study [16]. Few case reports showed recovery with an extended treatment of three months or more [18,35,37]. A case of chronic prostatitis treated for 3 months after the failure of an initial treatment of 6 weeks was also reported, which resulted in a clinical recovery with, however, the persistence of urinary colonization (patient 15 in Table 1).

As in the literature, we have found recurrence in 58% (7/12) of patients after 6 months of follow-up. This is probably partly due to the patient’s comorbidities. All of our patients with chronic prostatitis had an underlying urological disorder, which may explain recurrence of UTI. They also had a high Charlson comorbidity index, including immunosuppression in one third of patients, which may promote UTI. In addition, 25% (3/12) of the patients presented more than 1 new UTI during the follow-up, including 2 patients with several UTIs.

Despite negative urine culture follow-up, bacterial cells can persist in the form of a biofilm, which may induce the persistence of clinical signs. The presence of a bacterial biofilm represents a chronic inflammatory stimulus inducing the formation of prostatic calcifications that could lead to the persistence of symptoms related to the grade of inflammation and may be thought to favor infectious recurrences [38,39]. In our study, two patients had prostatic calcifications, of whom one relapsed within six months. In the series of Los Arcos et al., four out of the six patients with prostatic calcifications relapsed within six months [16]. Even with long-term antibiotherapy with FQ, which is the gold standard therapy, the recurrence rate in CBP is about 25–50% [40]; about 40% of recurrences are noticed at 6 months [41]. Thus, the recurrence rate in the case of treatment with oral FT does not appear to be major compared to a reference agent. It seems also to be partly explained by the patient’s comorbidities and its risk factors that are common to chronic prostatitis.

The European Committee on AST (EUCAST) defines Enterobacterales strains susceptibility to fosfomycin in the context of cystitis if the MIC is less than ≤32 mg/L [42]. Fosfomycin MIC was determined using Etest^®^ (BioMérieux, Marcy-L’Etoile, France) for four strains (3 *K. pneumoniae* and 1 *E. coli*). The 3 *K. pneumoniae* strains displayed fosfomycin MIC > 24 mg/L, while fosfomycin MIC for the *E. coli* isolate was 2 mg/L. Only one patient possessed an Enterobacterales strain displaying a high level of resistance to fosfomycin (MIC = 96 mg/L), which led to clinical and microbiological failure. It has been shown that fosfomycin MICs were significantly higher for *K. pneumoniae* (usually ≥4 mg/L) than *E. coli* (usually ≤4 mg/L) [32,43]. If the fosfomycin MIC is >4 mg/L, there is a real risk that intraprostatic concentrations will be below the MIC. Several authors advise against the use of fosfomycin in this situation [16,18,44,45]. Three of our patients possessed a strain with fosfomycin MICs > 4 mg/L, whereas the only patient in failure was infected with a strain for which the fosfomycin MIC was determined at 96 mg/L. We did not find any failure with a MIC between 16–32 mg/L. It is interesting to note that patient 10 had a MIC at 32 mg/L on the initial urine culture, and a controlled MIC at 16 mg/L on the urine culture during relapse, despite exposure to oral FT. The efficacy of fosfomycin despite high MICs has also been described by Oliveira Silva et al. with two cases of acute male *K. pneumoniae* UTI with MICs > 4 mg/L (8 and 128 mg/L) treated from 1 week but with a high dose of 3 g twice daily with good safety [19].

Thus, it seems important to determine fosfomycin MIC before treatment with oral fosfomycin in male UTIs, particularly in the case of *K. pneumoniae* strains. The result can possibly affect the administration frequency of this agent. For example, in case of poor safety of a daily FT treatment, a decrease of the FT dosage can be tried if the MIC is low. On the other hand, in the case of a high MIC, in order to increase the chances of having intra-prostatic concentrations at therapeutic doses, an increase in the dosage can be carried out according to safety.

These proposals concerning administration frequency are based on mainly pharmacokinetic data, but no clinical correlation has ever been demonstrated [18,20,36]. They should therefore be used with caution and, above all, according to the clinical presentation and the patient’s safety for the treatment.

In cases of chronic prostatitis, it is difficult to propose an optimal duration of treatment with oral FT. After a 6-week treatment, the risk of recurrence is around 50% and seems to reach the recurrence rate of other reference antibiotics, such as FQ (recurrences are usually between 25–50%) [40]. However, we reported one case of treatment being extended to three months with no recurrence within six months. Similarly, Karaiskos et al. proposed to treat for three months CBP with prostate calcification [17].

### 4.4. Fosfomycin-Trometamol Safety

In literature where treatment of acute male UTI was limited to three doses, there were few or no AEs. The absence of AEs was also found in 2 patients who received 6 g daily for 1 week [27,28,29,30]. In chronic prostatitis, Karaikos et al., with an oral FT regimen (similar to ours), showed an AE rate of 25% [34].

Diarrhea was the main side effect described. Patients presented diarrhea only when taking FT daily and not when taking FT every 48 h. Thus, the duration of treatment does not seem to increase the frequency of diarrhea, unlike the administration’s frequency. These data are consistent with studies of treatments of 3 months or longer where no adverse events were reported, except in one patient who received a double dose of 6 g per day for 5 days. [18,45]. In addition, pharmacokinetic dosage of oral fosfomycin in the long term shows stability of plasma concentration over time [18].

Finally, IV fosfomycin can usually be administered as a partner drug, as part of an antibiotic regimen, especially in patients with systemic involvement [46]. The presence of synergistic interactions, and the almost total absence of antagonisms, make fosfomycin a good alternative in combination regimens to treat KPC-, OXA-, and MBL-producing Enterobacterales and contribute to preventing antimicrobial resistance [46].

One of the limitations of our study was the retrospective methodology and also the limited number of patients. A randomized prospective study would be interesting to confirm and support our results.

## 5. Conclusions

In this era of the emergence of resistance to antimicrobial drugs, oral FT may have an important role in multi drug-resistant male UTIs. Our data suggest that oral FT could be safe and effective for use in chronic bacterial prostatitis, although over prolonged time periods.

## Figures and Tables

**Table 1:** Patient clinical and microbiological characteristics at baseline treated with oral fosfomycin-trometamol.

UTI	Patient/UTI Episodes	Age	Charlson Comorbidity Index/Immunosuppression	Urological Disorder/Creatinine * (umol/liter)	Clinical Features	CRP (mg/L)/Acute Kidney Failure	Bacteriuria/ ATS/ Fosfomycin MIC	Preliminary Antibiotics	Oral FT (Dosing, Duration)	Recovery (Clinical/ Microbiological)	Follow-Up ** (Recurrence/ITU New Episode)	Adverse Effects	Microbiological Colonization
**Acute UTI**	1	61	2/No	BPH***/88	Fever UFD	NR/83	*E. coli* 10^7^/ESBL, FQ R, SMX-TMP R/ND	FQ (2days)	3 g/24 h, 1 w	Yes/ND	0/0	Minor diarrhea	ND
	2	76	9/No	Non/56	Fever UFD, lower back pain	300/28	*E. coli* 10^6^/ESBL, FQ R, SMX-TMP R/ND	NTF (1day)	3 g/24 h, 3 w	Yes/Yes	0/0	No	No
	3	69	3/No	BPH/73	Fever UFD	160/60	*E. coli* 10^7^/ESBL, FQ R, SMX-TMP R/ND	FQ (2days) AMC (2days)	3 g/24 h, 3 w	Yes/Yes	0/0	No	No
	4	38	0/No	Non/90	UFD	<5/88	*K. pneumoniae* 10^5^/penicillinase, FQ R, SMX-TMP R/24	SMX-TMP (5days)	3 g/24 h, 3 w	Yes/Yes	0/0	Minor diarrhea	No
**Chronic bacterial prostatitis**	5	69	2/No	BPH, prostatic calcifications/86	UFD	170/74	*E. coli* 10^5^/penicillinase, FQ R, SMX-TMP R/ND	SMX-TMP (10days)	3 g/24 h, 3 w	Yes/Yes	0/0	Moderate diarrhea	No
	6/1st UTI	79	5/No	Prostatic adenocarcinoma/65	UFD	NR	*E. coli* 10^6^/ESBL, FQ R, SMX-TMP R/ND	No	3 g/24 h, 3 w	Yes/Yes	2 (M1, M3)/0	Non	No
	6/2nd UTI				UFD	NR	*E. coli* 10^6^/ESBL, FQ R, SMX-TMP R/ND	No	3 g/24 h, 3 w 3 g/48 h, 3 w	Yes/Yes	0/0	Minor diarrhea	No
	7	83	10/No	Prostatic adenocarcinoma/47	UFD	NR	*E. coli* 10^6^/ESBL, FQ R, SMX-TMP R/ND	No	3 g/24 h, 3 w	Yes/ND	0/0	Non	No
	8	68	10/No	BPH, prostatic calcifications/69	Fever UFD	NR	*E. coli* 10^7^/ESBL, FQ R, SMX-TMP R/ND	No	3 g/24 h, 2 w 3 g/48 h, 4 w	Yes/Yes	1 (M2)/0	Non	No
	9	76	7/Renal transplantation	Vesicoureteral reflux, HBP/36	UFD	20/33	*K. pneumoniae* 10^5^/ESBL, FQ R, SMX-TMP R/96	No	3 g/24 h, 3 w 3 g/48 h, 3 w	No/No	2 (W1, M3)/2 (M1, M2)	Non	No
	10	67	7/Cirrhosis	BPH, bladder disorders/95	UFD	40/104	*K. pneumoniae* 10^7^/ESBL, FQ R, SMX-TMP R/32	No	3 g/24 h, 3 w 3 g/48 h, 3 w	Yes/Yes	2 (M1, M4)/0	Non	Yes, (W1, MIC 16mg/L)
	11	53	2/Systemic Lupus Erythematosus	BPH/102	UFD	<5/102	*E. coli* 10^6^/ESBL, FQ R, SMX-TMP R/ND	No	3 g/24 h, 3 w 3 g/48 h, 3 w	Yes/Yes	0/0	Minor diarrhea	Non
	12/1st UTI	75	3/No	BPH/71	Fever UFD	<5/63	*E. coli* 10^5^/ESBL, FQ R, SMX-TMP R/ND	NTF (21days)	3 g/24 h, 3 w 3 g/48 h, 3 w	Yes/Yes	1 (W2)/0	Non	Non
	12/2nd UTI				UFD	NR	*E. coli* 10^5^/ESBL, FQ R, SMX-TMP R/ND	No	3 g/24 h, 3 w 3 g/48 h, 3 w	Yes/Yes	0/0	Non	Non
	13	45	3/No	Bladder disorders/105	Fever UFD	10/73	*E. coli* 10^6^/penicillinase, FQ R, SMX-TMP R/ND	SMX-TMP (7 days)	3 g/24 h, 3 w	Yes/ND	0/1 (M4)	Non	Non
	14	59	3/No	BPH, testicular implant/97	Fever UFD	NR/95	*E. coli* 10^5^/ESBL, FQ R, SMX-TMP R/ND	No	3 g/24 h, 3 w 3 g/48 h, 3 w	Yes/ND	0/2 (M2, M3)	Moderate diarrhea	Non
	15/1st UTI	71	3/No	BPH/79	Fever UFD	<5/81	*E. coli* 10^6^/FQ R, SMX-TMP R/ND	No	3 g/24 h, 2 w 3 g/48 h, 4 w	Yes/ND	1 (M6)/0	No	ND
	15/2nd UTI				Fever	NR	*E. coli* 10^6^/FQ R, SMX-TMP R/2	No	3 g/24 h, 3 w 3 g/48 h, 9 w	Yes/Yes	0/0	No	Yes (M3)
	16/1st UTI	69	6/Systemic Lupus Erythematosus, pulmonary transplantation	Urinary bladder carcinoma, bladder disorders/55	UFD	20/46	*K. pneumoniae* 10^7^/ESBL, FQ R, SMX-TMP R/ND	NTF (10 days)	3 g/24 h, 3 w 3 g/48 h, 3 w	Yes/ND	1 (M6)/0	No	NR
	16/2nd UTI				UFD	NR	*K. pneumoniae* 10^3^/ESBL, FQ R, SMX-TMP R/ND	No	3 g/24 h, 3 w 3 g/48 h, 3 w	Yes/Yes	1 (M3)/0	No	Non
	16/3rd UTI				UFD	NR	*K. pneumoniae* 10^5^/ESBL, FQ R, SMX-TMP R/ND	No	3 g/24 h, 1 w 3 g/48 h, 2 w	Yes/Yes	0/0	No	Non

Abbreviations: UTI: Urinary Tract Infection, BPH: Benign Prostate Hyperplasia, UFD: Urinary functional disorders, CRP: C-reactive protein, ATS: Antimicrobial Susceptibility Testing, MIC: Minimum Inhibitory Concentration, R: Resistence, ESBL: extended spectrum beta-lactamase, FQ: fluoroquinolones, SMX-TMP: trimethoprim/sulfamethoxazole, ND: not done, NTF: nitrofurantoin, AMC: amoxicillin-clavulanate, CFX: cefixime, FT: fosfomycin-trometamol, W: week, M: Month. * CKD (ml/min/1,73m^2^), ** Follow up M6: except for patient 4 (3M), patient 8 (4M), patient 12 UI 2 (4M), patient 16 UI 3 (1M).

**Table 2:** Treatment of Male Urinary Tract Infection with Oral fosfomycin-trometamol (review of literature).

UTI	Reference	Number/Number of Patients /Sex	Age	Comorbidities *	Clinical Features	Urine Culture/ATS	Fosfomycin MIC	Oral FT (Dosing)	Oral FT (Duration)	Microbiological Recovery	Clinical Recovery	Recurrence	Adverse Effects
**Acute UTI**	*Our study*	4/M	61	50% BPH, 25% CKD	Fever UFD	*75% E. coli, 25% K. pneumoniae/75%* ESBL, FQ R, SMX-TMP R	24 (1/4)	3 g/24 h	2.5 w	100% (4/4)	100% (4/4)	0% (0/4) (follow-up 5.3 M)	Diarrhea (2/4)
	*Da Silva, 2015* [19]	N°1/M	70	No	UFD	*K. pneumoniae*/PCE, FQ R	128	6 g/24 h	1 w	Yes	Yes	ND	No
		N°2/M	80	CKD	ND	*K. pneumoniae*/CPE, FQ R	8	6 g/24 h	1 w	Yes	Yes	ND	No
	*Neuner, 2012* [26]	19/M	NR	46% CKD 10% UT, 12% UD, 51% IS	UFD	Enterobacterale, *E. faecium*, *P. aeruginosa*/MDR	ND	2, 9 +/−1, 8 doses		58% (11/19)	ND	ND	ND
	*Qiao, 2013* [27]	105/M	NR	No	ND	NR	ND	3 g/48 h	5D (D1, D3, D5)	ND	77/105 (73%, w2)	ND	Diarrhea (5%)
	*Pullukcu, 2007* [28]	52/27 F (52%)	55 +/−18	10% UT, 8% bladder carcinoma, 10% IS	UFD	*E. coli*/ESBL, FQ R, SMX-TMP R	ND	3 g/24 h	3D	79% (41/52)	94% (49/52)	11% (3/28) (follow-up 1 M)	No
	*Senol, 2010* [29]	27/14 F (52%)	58 +/−15	19% CKD, 46% UT, 26% UD, 17% bladder carcinoma, 5% IS	UFD	*E. coli*/ESBL, FQ R, SMX-TMP R	ND	3 g/24 h	3D	59% (16/27)	78% (21/27)	6% (1/16) (follow-up 1 M)	No
	*Veve, 2016* [30]	89/66 F (74%)	69 +/−18	42% CKD, 9% IS, 38% UT	ND	84% *E. coli*, 15% *Klebsiella sp.*/BLSE, 95% FQ R, 80% SMX-TMP R	ND	62% 3 g/72 h 23% 3 g/48 h 13% 3 g/24 h	9D 6D 4.5D	13/89 patients consulted again within 30 days; 44% for clinical failure, 24% for recurrence, 8% for treatment adverse effects			
	*Nagel, 2015* [31]	43/35 F (81%)	63	Creatinine mean (umol/liter): 58 mL/min	UFD	25% Enterobacterales(16% EBLSE), 70% *Enterococcus* sp. (45% VRE), 5% others	ND	81% 3 g (1 dose) 19% 3 g/48 h	81% (1 dose) 19% 6D	95% (41/43)	ND	ND	NR
**Chronic bacterial prostatitis**	*Our study*	12/M	68	100% UD (67% BPH), 25% bladder carcinoma, 33% IS, 25% CKD	Fever (35%) UFD	*71% E. coli, 29% K. pneumoniae*/76% ESBL, 100% FQ R and SMX-TMP R	43 (3/17)	3 g/24-48 h	5.5 w	92% (11/12)	92% (11/12)	58% (7/12) (follow-up 5.8 M)	Diarrhea (25%, 3/12)
	*Grayson, 2015* [18]	N°1/M	73	No	Fever UFD	*E. coli*/ESBL, FQ R	1	3 g/24 h, (6 g/24 h, 5D)	16 w	Yes	Yes	No (follow-up 6 M)	Diarrhea (6 g/24 h)
		N°2/M	80	No	UFD	*E. coli*/ESBL, FQ R	1	3 g/24 h	12 w	Yes	Yes	No (follow-up 6 M)	No
	*Cunha, 2015* [32]	1/M (ITU 1)	53	BPH, prostatic calcifications	UFD	*E. coli*/ESBL, FQ R	2	3 g/72 h	4 w	ND	Yes	Yes (W1)	No
		1/M (ITU 2)			UFD	*E. coli*/ESBL, FQ R	ND	6 g/72 h	4 w	ND	Yes	Yes (W1)	NR
		1/M (ITU 3)			UFD	*E. coli*/ESBL, FQ R	2	3 g/72 h 2S + doxy + surgery		Yes	Yes	ND	NR
	*Karaikos, 2015* [33]	20/M	54	40% CKD	UFD	*E. coli* (13), *K. oxytoca* (3), others (4)/79% FQ R, 60% SMX-TMP R	32(median)	3 g/24 h 1S, puis 3 g/48 h	6 w	ND	85% (17/20)	ND	Diarrhea (25%)
	*Los-Arcos, 2016* [16]	15/M	54	7% CKD	UFD	*E. coli* (14), *K. oxytoca* (1)/26% BLSE, 66% FQ R, 33% SMX-TMP R	ND	77% 3 g/72 h 13% 3 g/48 h	6 w	ND	93% (14/15)	53% (8/15) à M20	No
	*Gian, 2016* [34]	1/M	53	BPH, prostatic calcifications	UFD	*Raoultella planticola*	ND	3 g/48 h	12 w	Yes	Yes	ND	NR
	*Matthews, 2016* [35]	4/M	79	1 Prostatic adenocarcinoma 1 CKD	ND	*E. coli*/ESBL	ND	50 doses		ND	4/4 (100%)	2/4 (50%)	No

Abbreviations: UTI: Urinary Tract Infection, BPH: Benign Prostate Hyperplasia, CKD: Chronic Kidney Disease, UT: urinary catheter (UT), UD: Urological Disorder, IS: immunosuppression, UFD: Urinary functional disorders, ND: Not Determined, ATS: Antimicrobial Susceptibility Testing, R: Resistance, ESBL: extended spectrum beta-lactamase, FQ: fluoroquinolones, SMX-TMP: trimethoprim/sulfamethoxazole, CPE: carbapenemase-producing Enterobacterales, MDR: multi-drug resistant, VRE: vancomycin-resistant Enterococcus, D: day, W: week, M: Month. * All patients were male except the studies 12, 13, 14 and 15 where the number of women was specified.

## Data Availability

Data available on request due privacy to restrictions. The data presented in this case study are available on request from the corresponding author.
